# Participation in Physical Activity is Associated with Sexual Activity in Older English Adults

**DOI:** 10.3390/ijerph16030489

**Published:** 2019-02-08

**Authors:** Lee Smith, Igor Grabovac, Lin Yang, Nicola Veronese, Ai Koyanagi, Sarah E. Jackson

**Affiliations:** 1 The Cambridge Centre for Sport and Exercise Sciences, Anglia Ruskin University, Cambridge CB1 1PT, UK; lee.smith@anglia.ac.uk; 2 Department of Social and Preventive Medicine, Centre for Public Health, Medical University Vienna, 1090 Vienna, Austria; 3 Department of Epidemiology, Center for Public Health, Kinderspitalgasse 15, 1st floor, 1090 Vienna, Austria; lin.yang@muv.ac.at; 4 National Research Council, Neuroscience Institute, Aging Branch, 35121 Padova, Italy; ilmannato@gmail.com; 5 Research and Development Unit, Parc Sanitari Sant Joan de Déu, Universitat de Barcelona, Fundació Sant Joan de Déu, 08950 Barcelona, Spain; koyanagi1117@hotmail.com; 6 Instituto de Salud Carlos III, Centro de Investigación Biomédica en Red de Salud Mental, CIBERSAM, 28029 Madrid, Spain; 7 Department of Behavioural Science and Health, University College London, London WC1E 7HB, UK; s.e.jackson@ucl.ac.uk

**Keywords:** sexual activity, sexual behaviour, sexual concerns, sexual problems, physical activity, television viewing time, older adults, England

## Abstract

Physical activity (PA) is a potential modifiable correlate of the age-related decline in sexual function, but no studies have explicitly tested this. This study aimed to examine associations between PA, television viewing (TV) time and sexual activity, problems, and concerns. Data were from 7038 men and women aged ≥50 years participating in the English Longitudinal Study of Ageing. PA and TV viewing time were self-reported. Sexual behaviour and concerns were assessed by self-completion questionnaire. Covariates included age, partnership status, socio-economic status, limiting long-standing illness, smoking status, alcohol intake and depressive symptoms. The odds of reporting any sexual activity were increased among individuals who participated in moderate (OR = 1.64, 95% CI: 1.24–2.15 in men) or vigorous (OR = 2.06, 95% CI: 1.50–2.84 in men, OR = 1.42, 95% CI: 1.09–1.85 in women) PA at least once a week. Erectile difficulties were less common among men who were active (OR = 0.58, 95% CI: 0.44–0.77 for vigorous PA). Women who watched ≥6 h of TV/day had lower odds of thinking about sex frequently (OR = 0.69, 95% CI: 0.50–0.96) or, if they did not live with a partner, being sexually active (OR = 0.40, 95% CI: 0.22–0.72). Encouraging older adults to be more physically active could help to improve sexual relationships and, as a result, mental health and wellbeing.

## 1. Introduction

An active sex life is associated with a plethora of mental and physical health benefits [1]. For example, intercourse frequency has been shown to predict greater quality of life [2], satisfaction with mental health [3], heart rate variability (an index that predicts cardiovascular health) [4], and lower risk of certain cancers [5,6] and fatal coronary events [7]. A healthy sex life may also improve life expectancy; in a study with a 25-year follow-up, greater frequency of sexual intercourse was associated with a lower annual death rate in men, and enjoyment of intercourse predicted lower mortality among women [8].

It is important to note that a decline in sexual activity has been found to be associated with psychophysiological factors. For example, a recent narrative review concluded that patients with erectile dysfunction are at increased risk of coronary heart disease (CHD) morbidity and/or mortality, as well as all-cause mortality [9]. This may be explained by the fact that vascular factors are a risk factor for erectile dysfunction as well as CHD. A recent meta-analysis also reported a high prevalence of erectile dysfunction among men with diabetes [10]. Erectile difficulties may also be caused by psychological factors relating to pre-diagnostic cancer symptoms, for example, stress, anxiety or depression [11]. It is plausible to assume that erectile dysfunction is associated with a decline in sexual activity, or at least intercourse. Finally, diminished pelvic blood flow secondary to aortoiliac or atherosclerotic disease leads to vaginal wall and clitoral smooth muscle fibrosis. This can ultimately result in symptoms of vaginal dryness and dyspareunia [12], which may result in a decrease in sexual desire.

Importantly, as people age, their risk of mental and physical health complications increases [13,14] and sexual activity declines. In a recent study of 3005 US middle-aged and older adults, the prevalence of any sexual activity was 73% among participants aged 57 to 64 years, 53% among those aged 65 to 74 years, and 26% among those aged 75 to 85 years [15]. In addition, the prevalence of sexual problems has been shown to increase with age. For example, globally it is estimated that by the time a man is in his 40s, he has about a 40% chance of having some form of erectile dysfunction, and this prevalence increases by around 10% per decade thereafter [16]. While less research has been carried out in women, the literature suggests that undiagnosed or untreated sexual problems, or both, can lead to or occur with depression or social withdrawal in middle or older aged men (40–70 years) [17,18]. Given the documented mental and physical health benefits of sexual activity across genders [19], it is important to identify modifiable correlates that may help prevent a decline in sexual activity in older adults and reduce sexual problems in this age group. 

One potential modifiable correlate is physical activity (PA). The rate of physical activity energy expenditure per unit time is commonly referred to as activity intensity. The rate of energy expenditure ranges from behaviours with very low energy expenditure, such as sleep, to vigorous activities with high energy expenditure, such as sprinting. This range is known as the energy expenditure continuum. Physical activity is the broad label that encompasses all movement of at least light intensity, and extends to movement of moderate and vigorous intensity. Regular and sustained participation in PA has been shown to be associated with higher levels of self-efficacy and physical functioning in older adults [20]. It is reasonable to assume that an increase in self-efficacy and physical function in older adults will facilitate greater sexual activity. Importantly, in men, regular and sustained physical activity—particularly aerobic exercise with moderate-to-vigorous intensity—has been shown to be associated with a lower prevalence of erectile dysfunction [21], a common problem in older adults. It is likely that less erectile dysfunction in older adults will lead to a more active sex life. 

At the other end of the energy expenditure continuum is sedentary behaviour. Sedentary behaviour is defined as any waking behaviour characterized by an energy expenditure ≤1.5 metabolic equivalents (METs) while in a sitting, reclining or lying posture [22]. Excessive sedentary time (≥6 h a day) has been shown to have an opposite influence on physical and mental health independent of physical activity. That is, it is associated with poorer mental health outcomes and worse physical health [23,24,25]. It is thus reasonable to assume that excessive sedentary time may be associated with less sexual activity and greater sexual problems. Television viewing (TV) time is a common proxy used for total sitting time and has been shown to be associated with a plethora of health outcomes (e.g., see [26,27,28,29]).

Therefore, the aim of the present study was to examine associations between physical activity, TV viewing time and sexual activity, problems, and concerns in a representative sample of older English adults. We hypothesized that higher levels of physical activity and lower levels of TV viewing time would be associated with higher levels of sexual activity, and fewer problems and concerns. 

## 2. Methods

### 2.1. Study Population

Data were drawn from the English Longitudinal Study of Ageing (ELSA), a longitudinal panel study of ageing, health, and wellbeing among men and women aged ≥50 years living in private households in England [30]. The Sexual Relationships and Activities Questionnaire (SRA-Q) was administered as a self-completion measure in wave 6 (2012/13) and was returned by 7079 participants (67% of those eligible). Our analyses use these data in addition to data on physical activity, TV viewing time and sociodemographic and health-related covariates also assessed in wave 6. We excluded 40 individuals who failed to state whether or not they had been sexually active over the last year, and one who had missing data on physical activity, leaving a final analytical sample of 7038 men and women. All participants gave their informed consent for inclusion before they participated in the study. The study was conducted in accordance with the Declaration of Helsinki, and the protocol was approved by the London Multi-Centre Research Ethics Committee.

### 2.2. Measurement of Exposures

Physical activity was assessed with three items that asked participants how often they took part in vigorous, moderate and low-intensity activities (more than once a week, once a week, 1–3 times a month, hardly ever/never) [31]. Physical activity was further categorised into three categories, as previously described [32]: inactive (no moderate/vigorous activity on a weekly basis), moderate activity at least once a week, and vigorous activity at least once a week.

TV viewing time was assessed with two questions about TV viewing: “How many hours of television do you watch on an ordinary day or evening, that is, Monday to Friday?” and “How many hours of television do you normally watch in total over the weekend, that is, Saturday and Sunday?” Average daily time spent watching TV was calculated as [(weekday TV time × 5) + (weekend TV time)]/7]. Daily TV time was categorised into four groups (<2hours/day; 2 to <4 h/day; 4 to <6hours/day; ≥6 h/day) [33].

### 2.3. Measurement of Outcomes

For outcomes measurement we used the SRA-Q, for which more information is available elsewhere [34]. The SRA-Q was derived from previously validated measures with modifications to ensure comparability with the National Social Life, Health and Aging Study in the USA [35] and with the National Survey of Sexual Attitudes and Lifestyles in the UK [36]. It captures a wide range of information on attitudes to sex, frequency of sexual activities and behaviours, problems with sexual activities and function, concerns and worries about sexual activities and sexual function, and sexual satisfaction. The male and female versions of the SRA-Q are available online at http://www.elsa-project.ac.uk/documentation. Participants completed the questionnaire in private and returned it in a sealed envelope. Details of the items presented in this report are provided in the online supplementary material. For the purposes of our study, we specifically looked into the frequency of sexual activity, namely, frequent (defined as ≥2 times per month) sexual intercourse, frequent kissing, petting and fondling, as well as combined. Given the differences in questions aimed at men and women in SRA-Q, we stratified the sample based on gender and provide results for men and women separately. 

### 2.4. Measurement of Potential Confounders

All potential confounders were selected *a priori* on the basis that they were likely to be related both to level of physical activity and sexual outcomes. Demographic information collected included age, gender, and partnership status (married/cohabiting, separated/divorced, widowed, or single/never married). Socio-economic status was based on household non-pension wealth, because it has been identified as particularly relevant to health outcomes in this age group [37], categorised into quintiles across all ELSA participants who took part in wave 6. Health-related questions included self-reported limiting long-standing illness (defined as any long-standing illness, disability or infirmity that limits activities in any way), current smoking status (smoker or non-smoker) and frequency of alcohol intake, categorised as never/rarely (never—once or twice a year), regularly (once every couple of months—twice a week), or frequently (3 days a week—almost every day) [34]. Depressive symptoms were assessed using the 8-item Centre of Epidemiological Studies Depression (CES-D) scale, which is highly validated for use in older adults [38].

### 2.5. Statistical Analysis

Analyses were performed using IBM SPSS Statistics 22. Data were weighted to correct for sampling probabilities and for differential non-response and to calibrate back to the 2011 National Census population distributions for age and sex. The weights accounted for the differential probability of being included in wave 6 of ELSA and for non-response to the SRA-Q. Details can be found at http://doc.ukdataservice.ac.uk/doc/5050/mrdoc/pdf/5050_elsa_w6_technical_report_v1.pdf.

We used logistic regression to analyse associations between level of physical activity and TV viewing time with sexual activities and concerns, with age, partnership status, wealth, limiting long-standing illness, smoking status, alcohol intake and depressive symptoms as covariates. Separate analyses were carried out on men and women, with variables relating to the frequency of sexual activity carried out in the full sample and separately for participants who reported living/not living with a partner. The results are presented as age-adjusted percentages and adjusted odds ratios (ORs), with 95% confidence intervals (CIs). For analyses of physical activity, the inactive group was the reference category. For analyses of TV viewing time, <2 h/day was the reference category.

### 2.6. Patient Involvement

No patients were involved in setting the research question or the outcome measures, nor were they involved in the design and implementation of the study. There are no plans to involve patients in dissemination.

## 3. Results

There were 3112 men and 3926 women in the study. Characteristics of the male and female samples are shown in Table 1. 

### 3.1. Sexual Activity and Physical Activity

The majority of men (77.7%) and women (53.8%) were sexually active, defined as reporting any sexual activity (including vaginal, anal or oral intercourse or other sexual activities such as kissing, petting or fondling) within the last year. However, fewer men who were inactive (64.6%) than who were moderately (79.5%) or vigorously active (83.4%) reported any sexual activity over the past year (Table 2). After adjustment for covariates, moderate PA at least once a week was independently associated with a 64% (95% CI: 24%–115%) increase in the odds of reporting any sexual activity, and vigorous PA at least once a week was associated with a 106% (95% CI: 50%–184%) increase. A similar pattern was seen in women, with those who were inactive (43.6%) less likely to report sexual activity than those who were moderately (56.5%) or vigorously active (65.4%) over the past year (Table 3). In women, vigorous PA at least once a week was associated with a 42% (95% CI: 9%–85%) increase in the odds of reporting sexual activity. The difference between inactivity and moderate PA engagement in women was only significant in those not living with a partner. Physical activity was independently associated with higher rates of thinking about sex in women (OR = 1.37, 95% CI: 1.10–1.72 moderate PA, OR = 1.80, 95% CI: 1.39–2.35 vigorous PA) compared to those inactive, but not in men.

Among sexually active men, we observed no differences in the frequency of sexual activity by level of physical activity. Among sexually active women, there was no association between physical activity and the frequency of sexual intercourse, but women who were moderately active had reduced odds of reporting frequent kissing, petting or fondling (OR = 0.58, 95% CI: 0.42–0.80) relative to those who were inactive (Table 3).

Erectile difficulties were more common among inactive men (51.3%) compared with those who were moderately (40.8%) and vigorously active (33.0%). After adjusting for covariates, vigorous PA at least once a week was independently associated with a 42% (95% CI: 33%–66%) reduction in the odds of erectile difficulties. Difficulty becoming sexually aroused was more common among inactive women (35.4%) compared with those who were moderately (32.5%) and vigorously active (29.2%), but differences were not significant.

### 3.2. Sexual Concerns and Physical Activity

There were no significant differences by level of physical activity in concerns about ability to become sexually aroused or have an erection or orgasmic experience. However, men who were moderately (OR = 1.79, 95% CI: 1.07–2.99) or vigorously active (OR = 1.72, 95% CI: 1.00–2.94) were more likely to cite concerns about the frequency of sexual activities (Table 2), and women who were vigorously active were more concerned about their level of sexual desire (OR = 2.09, 95% CI: 1.14–3.82) (Table 3). Men who were vigorously active were more likely to report dissatisfaction with their overall sexual activity (OR = 1.64, 95% CI: 1.02–2.65).

### 3.3. Sexual Activity and TV Viewing Time

Men who watched between 2 and 4 h of TV a day had significantly higher odds of reporting any sexual activity (OR = 1.61, 95% CI: 1.08–2.41), thinking about sex frequently (OR = 1.75, 95% CI: 1.16–2.63) and frequent sexual intercourse (OR = 1.44, 95% CI: 1.08–1.93) than those who watched <2 h a day, although these differences were not observed for those who watched more than 4 h of TV a day (Table 4). Men who watched more than 2 h of TV a day had increased odds of reporting frequent kissing, petting or fondling (≥6 h/day TV viewing, OR = 1.56, 95% CI: 1.13–2.17, *p* for trend = 0.030). Among women, TV viewing time was not significantly associated with sexual activity overall, although in those who were not living with a partner watching ≥6 h of TV a day was associated with reduced odds of being sexually active (OR = 0.40, 95% CI: 0.22–0.72) (Table 5). Women who watched the most TV were also less likely to report thinking about sex frequently (OR = 0.69, 95% CI: 0.50–0.96). We observed no significant associations between TV viewing time and measures of sexual functioning in either men or women.

### 3.4. Sexual Concerns and TV Viewing Time

Among men, there was no significant association between TV viewing time and concerns about sexual activity or function, although men who watched ≥6 h of TV a day were significantly less likely to report dissatisfaction with their overall sexual activity (OR = 0.58, 95% CI: 0.35–0.97) (Table 4). However, women who watched more TV were significantly less concerned about their level of sexual desire (≥6 h/day TV viewing, OR = 0.44, 95% CI: 0.26–0.75, *p* for trend = 0.001) and orgasmic experience (≥6 h/day TV viewing, OR = 0.44, 95% CI: 0.22–0.87, *p* for trend = 0.013), and were slightly less dissatisfied with their sexual activity (Table 5).

## 4. Discussion

In this large representative sample of older English adults, men and women who participated in physical activity at least once a week were significantly more likely to report any sexual activity in the last year and were less likely to report sexual problems. It is likely that those older adults who are physically active have better physical function and higher levels of self-efficacy for performing physical tasks than those who are inactive [20], which may lead to increased frequency of sexual activity; however, this hypothesis remains untested and future research is required. Effects of physical activity on energy expenditure mean active individuals are also likely to have a lower body mass index, which has been shown to be associated with more frequent sexual activity in this age group [39]. The finding that those who are physically active have fewer sexual problems is consistent with previous research linking physical activity to better sexual functioning [40,41]. Differences may be due to active individuals having better cardiovascular health, and vascular function, and fewer metabolic disturbances compared with those who are inactive, at least in the case of erectile dysfunction [21,42], and likely contribute to greater sexual activity in older adults.

Interestingly, despite reporting higher rates of sexual activity and fewer problems with sexual functioning, those who were physically active were more likely to report concerns about their sex lives. Specifically, physically active men were concerned about the frequency of their sexual activity. It may be that men who participate in high levels of physical activity have a higher “sex drive” than those who take part in low levels. Therefore, those with high levels of activity are likely to be less satisfied with the same frequency of sex when compared to those with low levels. However, it should be noted that this hypothesis remains untested. Women were most concerned about their level of sexual desire (although the direction of concern, i.e., too high or too low, was not specified). This finding is likely owing to a higher prevalence of sexual activity in the physically active group compared to those who are inactive. Those who are inactive are less likely to be sexually active and thus less likely to have concerns about their sexual desire.

Few differences were found between high and low levels of TV viewing and sexual outcomes, suggesting that an increase in physical activity needs to be adopted rather than a reduction in TV viewing time. Indeed, the present study found that in women watching high amounts of TV were less dissatisfied with their sexual activity. It may be that TV viewing with a partner provides a setting for intimacy.

As people age, levels of physical activity tend to decline. A previous study in the ELSA cohort observed low levels of physical activity among older adults and an overall trend for increasing levels of inactivity and a reduction in vigorous activity with age [43]. It is likely that low and declining physical activity levels in the older population contribute, at least in part, to the established age-related decline in sexual activity [17] and increase in sexual problems [18]. Interventions are needed to promote physical activity in older adults. Such an intervention may aim to promote such activities of a moderate intensity that do not necessarily require high levels of functional fitness (e.g., brisk walking); such interventions may yield long-lasting effects.

This is the first study to investigate the relationship between levels of physical activity, TV viewing time and sexual activity, problems, and concerns. The strengths of the study include the large representative sample of older English adults, and adjustment for a range of sociodemographic and health-related confounders. However, the findings must be interpreted considering the limitations of the study. The cross-sectional study design precludes causal inferences from being made. It is therefore not known whether being physically active leads to greater sexual activity and fewer sexual problems or vice versa, although it seems less plausible that sexual activity is driving physical activity. A further limitation is the self-reported measure of physical activity used in the present study. Participants may over report physical activity in fear of being judged. However, the self-reported physical activity variable has previously been shown to be moderately correlated with objectively assessed hours per day of moderate-vigorous intensity activity (Spearman’s r = 0.21, *p* = 0.020) [44].

## 5. Conclusions

In conclusion, findings from the present study suggest for the first time that participating in physical activity during later life may facilitate a more active and less troublesome sexual activities. Accordingly, they provide support for the promotion of physical activity in older adults to improve sexual activity. In addition, with growing evidence indicating that sexual activity may protect against a range of mental and physical health problems, these findings point to a novel pathway through which physical activity may promote health and wellbeing in older age.

## Figures and Tables

**Table 1:** Sample characteristics.

	Men (*n* = 3112)	Women (*n* = 3926)
Age (mean [SD] years)	64.37 (9.82)	65.29 (10.15)
Partner status		
Married/cohabiting	73.2	59.5
Separated/divorced	11.2	15.5
Widowed	6.9	19.1
Single/never married	8.7	5.9
Wealth quintile		
1 (poorest)	17.4	20.7
2	19.1	20.8
3	19.6	21.0
4	22.3	19.2
5 (richest)	21.6	18.4
Limiting long-standing illness		
Yes	31.8	36.6
No	68.2	63.4
Smoking status		
Smoker	14.5	13.5
Non-smoker	85.5	86.5
Alcohol intake ^1^		
Never/rarely	16.1	30.6
Regularly	41.7	43.1
Frequently	42.2	26.3
Depressive symptoms (mean [SD])	1.15 (1.82)	1.59 (2.03)
Physical activity		
Inactive	20.2	27.0
Moderately active at least once a week	43.1	46.8
Vigorously active at least once a week	36.7	26.1
TV viewing time		
<2 h/day	13.6	10.3
2 < 4 h/day	32.8	28.1
4 < 6 h/day	26.4	27.9
≥6 h/day	27.3	33.7

Values are percentages unless otherwise stated. All figures are weighted for sampling probabilities and differential non-response. SD = standard deviation. ^1^ Never/rarely = never—once or twice a year; regularly = once every couple of months—twice a week; and frequently = 3 days a week—almost every day.

**Table 2:** Sexual activities, functioning and concerns in relation to physical activity—men.

	Age-Adjusted Percentages			Adjusted OR [95% CI]	
	(1) Inactive	(2) Moderate PA at Least Once a Week	(3) Vigorous PA at Least Once a Week	(2) vs. (1)	(3) vs. (1)
**Sexual activity and behaviour**					
Any sexual activity in the past year					
All	64.6	79.5	83.4	1.64 [1.24–2.15] ***	2.06 [1.50–2.84] ***
Living with a partner	67.5	82.1	84.8	1.90 [1.36–2.65] ***	2.32 [1.60–3.37] ***
Not living with a partner	58.0	69.5	77.7	1.22 [0.76–1.97]	1.74 [0.90–3.36]
Thinking about sex frequently	72.5	81.7	83.5	1.12 [0.84–1.48]	1.10 [0.79–1.52]
Frequent ^1^ sexual intercourse †					
All	40.7	46.4	52.2	0.97 [0.73–1.29]	1.09 [0.81–1.47]
Living with a partner	47.6	50.1	52.8	0.92 [0.66–1.28]	0.94 [0.67–1.32]
Not living with a partner	26.0	28.6	49.3	0.84 [0.47–1.52]	1.66 [0.88–3.13]
Frequent ^1^ kissing, petting, or fondling †					
All	53.2	63.1	68.2	1.13 [0.86–1.49]	1.31 [0.98–1.75]
Living with a partner	64.1	68.2	70.7	1.00 [0.72–1.40]	1.12 [0.79–1.59]
Not living with a partner	28.7	39.5	55.2	1.23 [0.70–2.17]	1.67 [0.89–3.13]
**Sexual function**					
Erectile difficulties	51.3	40.8	33.0	0.80 [0.62–1.04]	0.58 [0.44–0.77] ***
Difficulty achieving orgasm ‡	17.8	14.7	11.1	0.99 [0.61–1.58]	0.72 [0.43–1.20]
**Sexual health concerns and satisfaction**					
Concerned about…					
Level of sexual desire	17.1	16.2	12.5	1.16 [0.75–1.78]	0.95 [0.60–1.50]
Frequency of sexual activities †	10.3	13.8	11.9	1.79 [1.07–2.99] *	1.72 [1.00–2.94] *
Ability to have an erection	20.2	14.6	11.9	0.94 [0.61–1.44]	0.82 [0.52–1.30]
Orgasmic experience ‡	14.6	11.2	9.2	0.91 [0.54–1.51]	0.75 [0.43–1.29]
**Dissatisfied with overall sexual activity ¥**	17.5	20.4	20.8	1.49 [0.94–2.36]	1.64 [1.02–2.65] *

^1^ ≥2 times per month. † In participants reporting any sexual activity in the past year. ‡ In participants reporting any sexual activity in the past month. ¥ In participants reporting any sexual activity with a partner in the past 3 months. OR odds ratio; CI confidence interval. Odds ratios are adjusted for age, wealth quintile, limiting long-standing illness, smoking status, alcohol, intake and depressive symptoms. * *p* < 0.05, *** *p* < 0.001. All analyses are weighted for sampling probabilities and differential non-responses.

**Table 3:** Sexual activities, functioning and concerns in relation to physical activity—women.

	Age-Adjusted percentages			Adjusted OR [95% CI]	
	(1) Inactive	(2) Moderate PA at Least Once a Week	(3) Vigorous PA at Least Once a Week	(2) vs. (1)	(3) vs. (1)
**Sexual activity and behaviour**					
Any sexual activity in the past year					
All	43.6	56.5	65.4	1.21 [0.97–1.52]	1.42 [1.09–1.85] *
Living with a partner	61.6	72.0	75.3	1.19 [0.90–1.57]	1.15 [0.82–0.62]
Not living with a partner	16.7	25.4	41.5	1.77 [1.12–2.79] *	3.33 [1.98–5.60] ***
Thinking about sex frequently	36.8	47.4	57.3	1.37 [1.10–1.72] **	1.80 [1.39–2.35] ***
Frequent sexual intercourse†					
All	45.0	48.7	53.8	0.88 [0.65–1.19]	1.03 [0.75–1.43]
Living with a partner	48.9	51.0	56.1	0.89 [0.64–1.23]	1.03 [0.72–1.48]
Not living with a partner	24.1	36.4	41.6	1.35 [0.56–3.23]	1.82 [0.73–4.51]
Frequent kissing, petting, or fondling †					
All	69.3	64.3	71.6	0.58 [0.42–0.80] **	0.75 [0.52–1.07]
Living with a partner	75.5	69.2	75.1	0.60 [0.42–0.87] **	0.72 [0.48–1.09]
Not living with a partner	35.6	37.2	52.8	0.76 [0.34–1.71]	1.70 [0.73–3.95]
**Sexual function**					
Difficulty becoming sexually aroused ‡	35.4	32.5	29.2	1.02 [0.70–1.49]	0.85 [0.56–1.28]
Difficulty achieving orgasm ‡	30.1	27.5	28.4	0.98 [0.67–1.44]	1.13 [0.74–1.71]
**Sexual health concerns and satisfaction**					
Concerned about…					
Level of sexual desire	10.1	10.9	12.1	1.63 [0.92–2.88]	2.09 [1.14–3.82] *
Frequency of sexual activities †	7.5	6.7	9.9	1.12 [0.59–2.13]	1.91 [0.99–3.68]
Ability to become sexually aroused ‡	9.5	7.0	7.3	0.98 [0.52–1.82]	0.95 [0.47–1.90]
Orgasmic experience ‡	8.6	6.4	7.9	1.16 [0.59–2.27]	1.49 [0.72–3.06]
**Dissatisfied with overall sexual activity ¥**	9.7	12.8	12.5	1.63 [0.90–2.94]	1.78 [0.95–3.34]

† In participants reporting any sexual activity in the past year. ‡ In participants reporting any sexual activity in the past month. ¥ In participants reporting any sexual activity with a partner in the past 3 months. OR odds ratio; CI confidence interval. Odds ratios are adjusted for age, wealth quintile, limiting long-standing illness, smoking status, alcohol intake, and depressive symptoms. All analyses are weighted for sampling probabilities and differential non-responses. * *p* < 0.05, ** *p* < 0.01, *** *p* < 0.001.

**Table 4:** Sexual activities, functioning and concerns in relation to hours of daily TV viewing—men.

	Age-Adjusted Percentages				Adjusted OR [95% CI]		
	(1) <2 h/day	(2) 2 < 4 h/day	(3) 4 < 6 h/day	(4) ≥6 h/day	(2) vs. (1)	(3) vs. (1)	(4) vs. (1)
**Sexual activity and behaviour**							
Any sexual activity in the past year							
All	77.6	83.8	77.3	72.3	1.61 [1.08–2.41] *	1.02 [0.69–1.51]	0.98 [0.67–1.45]
Living with a partner	81.6	85.5	78.9	75.9	1.59 [0.97–2.59]	0.93 [0.58–1.51]	0.95 [0.59–1.55]
Not living with a partner	66.5	76.1	70.9	61.6	1.56 [0.76–3.16]	1.23 [0.62–2.44]	1.02 [0.53–1.96]
Thinking about sex frequently	80.2	86.4	79.7	74.5	1.75 [1.16–2.63] **	0.94 [0.63–1.40]	0.87 [0.59–1.29]
Frequent sexual intercourse†							
All	43.6	53.7	47.4	42.4	1.44 [1.08–1.93] *	1.19 [0.88–1.62]	1.12 [0.81–1.55]
Living with a partner	46.9	56.3	49.3	46.0	1.45 [1.04–2.01] *	1.07 [0.76–1.51]	1.05 [0.73–1.53]
Not living with a partner	31.9	38.2	38.2	32.0	1.00 [0.49–2.03]	1.39 [0.67–2.87]	1.34 [0.65–2.74]
Frequent kissing, petting, or fondling †							
All	57.9	68.0	64.1	60.6	1.51 [1.12–2.03] **	1.52 [1.11–2.08] **	1.56 [1.13–2.17] **
Living with a partner	63.5	72.1	68.7	66.4	1.43 [1.02–2.00] *	1.35 [0.94–1.93]	1.34 [0.92–1.96]
Not living with a partner	38.4	44.8	43.3	42.0	1.12 [0.56–2.25]	1.55 [0.75–3.18]	2.00 [0.98–4.09]
**Sexual function**							
Erectile difficulties	38.6	35.2	41.1	45.0	0.84 [0.61–1.16]	1.04 [0.75–1.44]	1.07 [0.77–1.49]
Difficulty achieving orgasm ‡	11.9	13.4	14.1	14.0	1.08 [0.62–1.91]	1.06 [0.58–1.92]	0.99 [0.53–1.83]
**Sexual health concerns and satisfaction**							
Concerned about…							
Level of sexual desire	14.3	13.8	15.6	15.3	0.77 [0.49–1.21]	0.87 [0.54–1.40]	0.73 [0.44–1.19]
Frequency of sexual activities †	13.8	10.2	14.1	13.8	0.69 [0.43–1.11]	0.97 [0.59–1.60]	0.84 [0.50–1.41]
Ability to have an erection	12.6	12.0	15.5	17.3	0.76 [0.46–1.25]	1.02 [0.60–1.71]	0.94 [0.55–1.61]
Orgasmic experience ‡	10.5	9.3	12.0	11.9	0.71 [0.41–1.25]	0.97 [0.54–1.74]	0.85 [0.46–1.57]
**Dissatisfied with overall sexual activity ¥**	20.7	19.7	25.5	14.5	0.85 [0.56–1.30]	1.24 [0.79–1.93]	0.58 [0.35–0.97] *

† In participants reporting any sexual activity in the past year. ‡ In participants reporting any sexual activity in the past month. ¥In participants reporting any sexual activity with a partner in the past 3 months. OR odds ratio; CI confidence interval. Odds ratios are adjusted for age, wealth quintile, limiting long-standing illness, smoking status, alcohol intake, and depressive symptoms. All analyses are weighted for sampling probabilities and differential non-responses.* *p* < 0.05, ** *p* < 0.01.

**Table 5:** Sexual activities, functioning and concerns in relation to hours of daily TV viewing—women.

	Age-Adjusted Percentages				Adjusted OR [95% CI]		
	(1) <2 h/day	(2) 2 < 4 h/day	(3) 4 < 6 h/day	(4) ≥6 h/day	(2) vs. (1)	(3) vs. (1)	(4) vs. (1)
**Sexual activity and behaviour**							
Any sexual activity in the past year							
All	58.9	62.1	57.8	47.3	0.96 [0.68–1.35]	1.01 [0.72–1.43]	0.75 [0.53–1.05]
Living with a partner	72.5	77.0	70.8	64.3	1.15 [0.74–1.79]	0.93 [0.60–1.44]	0.74 [0.48–1.15]
Not living with a partner	34.1	28.2	30.0	18.2	0.57 [0.32–1.03]	0.83 [0.46–1.49]	0.40 [0.22–0.72] **
Thinking about sex frequently	51.9	54.0	49.9	38.4	1.09 [0.79–1.52]	1.05 [0.75–1.46]	0.69 [0.50–0.96] *
Frequent sexual intercourse†							
All	55.1	51.6	48.4	47.0	0.94 [0.66–1.34]	0.79 [0.55–1.15]	0.83 [0.58–1.21]
Living with a partner	57.7	53.2	51.2	50.5	0.84 [0.57–1.26]	0.76 [0.50–1.15]	0.80 [0.52–1.21]
Not living with a partner	43.6	40.7	35.3	28.0	1.08 [0.46–2.52]	0.74 [0.31–1.77]	0.53 [0.21–1.37]
Frequent kissing, petting, or fondling †							
All	70.2	73.0	64.5	63.3	1.09 [0.74–1.61]	0.70 [0.47–1.04]	0.82 [0.55–1.22]
Living with a partner	75.5	77.7	69.4	67.2	0.98 [0.61–1.57]	0.60 [0.38–0.98] *	0.63 [0.39–1.02]
Not living with a partner	48.1	42.9	40.6	40.7	0.75 [0.33–1.72]	0.61 [0.26–1.42]	0.78 [0.32–1.89]
**Sexual function**							
Difficulty becoming sexually aroused ‡	31.0	29.1	32.7	34.9	0.84 [0.54–1.33]	1.25 [0.78–1.98]	1.33 [0.83–2.12]
Difficulty achieving orgasm ‡	32.8	26.1	28.8	28.5	0.68 [0.44–1.05]	0.84 [0.54–1.33]	0.75 [0.47–1.19]
**Sexual health concerns and satisfaction**							
Concerned about…							
Level of sexual desire	19.7	8.6	9.4	12.7	0.35 [0.21–0.59] ***	0.42 [0.25–0.72] **	0.44 [0.26–0.75] **
Frequency of sexual activities †	9.8	6.9	7.9	8.6	0.78 [0.41–1.48]	0.99 [0.51–1.92]	0.87 [0.44–1.71]
Ability to become sexually aroused ‡	6.0	5.9	7.8	10.0	0.88 [0.39–1.98]	1.51 [0.67–3.41]	1.54 [0.68–3.49]
Orgasmic experience ‡	13.7	6.5	5.1	7.7	0.41 [0.22–0.78] **	0.33 [0.16–0.69] **	0.44 [0.22–0.87] *
**Dissatisfied with overall sexual activity ¥**	15.7	10.9	12.2	12.4	0.53 [0.31–0.91] *	0.65 [0.37–1.15]	0.65 [0.36–1.15]

† In participants reporting any sexual activity in the past year. ‡ In participants reporting any sexual activity in the past month. ¥ In participants reporting any sexual activity with a partner in the past 3 months. OR odds ratio; CI confidence interval. Odds ratios are adjusted for age, wealth quintile, limiting long-standing illness, smoking status, alcohol intake, and depressive symptoms. All analyses are weighted for sampling probabilities and differential non-responses. * *p* < 0.05, ** *p* < 0.01, *** *p* < 0.001.
